# How the ESRF helps industry and how they help the ESRF

**DOI:** 10.1107/S0907444913001108

**Published:** 2013-06-18

**Authors:** Stéphanie Malbet-Monaco, Gordon A. Leonard, Edward P. Mitchell, Elspeth J. Gordon

**Affiliations:** a Structural Biology Group, European Synchrotron Radiation Facility, 6 Rue Jules Horowitz, 38043 Grenoble, France; b Business Development Office, European Synchrotron Radiation Facility, 6 Rue Jules Horowitz, 38043 Grenoble, France

**Keywords:** synchrotron MX beamlines, proprietary access, service data collection, automation

## Abstract

The ESRF has worked with, and provided services for, the pharmaceutical industry since the construction of its first protein crystallography beamline in the mid-1990s. In more recent times, industrial clients have benefited from a portfolio of beamlines which offer a wide range of functionality and beam characteristics, including tunability, microfocus and micro-aperture. Included in this portfolio is a small-angle X-­ray scattering beamline dedicated to the study of biological molecules in solution. The high demands on throughput and efficiency made by the ESRF’s industrial clients have been a major driving force in the evolution of the ESRF’s macromolecular crystallography resources, which now include remote access, the automation of crystal screening and data collection, and a beamline database allowing sample tracking, experiment reporting and real-time at-a-distance monitoring of experiments. This paper describes the key features of the functionality put in place on the ESRF structural biology beamlines and outlines the major advantages of the interaction of the ESRF with the pharmaceutical industry.

## Abstract 2

The key features of the functionality facilitating proprietary use of the ESRF’s structural biology beamlines are described, as are the major advantages, in terms of beamline evolution, of the interaction of the ESRF with the pharmaceutical industry.

## 1. Introduction

Industrial users have accessed the facilities available at the European Synchrotron Radiation Facility (ESRF) for a wide range of experiments requiring the use of synchrotron radiation (SR) since the start of user operation in 1994. Proprietary access consists mainly of beam-time sales and related services and is dominated by activity in two fields: macromolecular crystallography (MX; used by the drug-discovery programmes of pharmaceutical and biotechnology companies) and micro-tomography (used by a very wide range of industrial sectors). The income generated from industrial activity at the ESRF is reinvested into improving the existing facilities and providing extra staff. Access by clients from the pharmaceutical industry is centred on the MX beamlines ID14 (Wakatsuki *et al.*, 1998 ▶; McCarthy *et al.*, 2009 ▶), ID23 (Nurizzo *et al.*, 2006 ▶; Flot *et al.*, 2010 ▶) and ID29 (de Sanctis *et al.*, 2012 ▶), and currently accounts for around 50% of ESRF beam-time sales (Mitchell *et al.*, 2011 ▶). Industrial clients of the MX beamlines can purchase beam time and perform their experiments themselves either at the ESRF or *via* remote access from their home laboratory. Alternatively, they can take advantage of the MXpress data-collection service. Here, cryocooled samples are shipped to the ESRF, where data collection is carried out by ESRF staff.

In order to optimize the service offered to all users of the ESRF’s structural biology beamlines, and to simplify support issues, the ESRF has embarked upon a process of automation and standardization (Leonard *et al.*, 2007 ▶; Arzt *et al.*, 2005 ▶). The high-throughput demands of proprietary researchers and their willingness to adopt automatic procedures have played a major part in driving and debugging this process. Experiences with industrial researchers have helped in the development of a reliable robotic sample changer (Cipriani *et al.*, 2006 ▶), an automatic data-processing pipeline (Monaco *et al.*, 2012 ▶), remote-access experiments (Gabadinho *et al.*, 2010 ▶) and a laboratory information management system (LIMS), ISPyB (Beteva *et al.*, 2006 ▶; Delagenière *et al.*, 2011 ▶). The latter combines the sample tracking and experiment reporting essential for the running of an efficient data-collection service for proprietary clients.

Many of the lessons that have been learnt from automation of the MX beamlines can be used to automate/streamline other synchrotron experiments. Indeed, pipelines for the automatic collection and treatment of small-angle X-­ray scattering data from solutions of biological macromolecules (BioSAXS) and a LIMS for experiments on BioSAXS beamlines (Pernot *et al.*, 2010 ▶) are currently under construction in collaboration with Diamond Light Source and the EMBL Hamburg Outstation. This paper describes the impetus given to the automation of the ESRF MX beamlines as a result of proprietary research and summarizes the key features of the developments put in place at the ESRF from which all users of the beamlines have benefited.

## 2. Macromolecular crystallography and drug discovery

MX in the pharmaceutical development setting can take several forms (Congreve *et al.*, 2005 ▶) and is often used in concert with other biophysical techniques in the discovery of drug lead candidates, lead optimization, profiling of clinical resistance and understanding of selectivity and potency. Once a lead candidate has been selected, a cocrystal structure with the target molecule can reveal detailed information on binding modes which is often critical for further design ideas and for the prioritization of these design ideas. Once the structural biology design process has been undertaken then regular access to structural information and input from medicinal chemists is required to make the procedure work.

Perhaps the greatest impact of SR in the pharmaceutical setting has been, in the early stages of drug discovery, the adoption of high-throughput crystallography (HTX) in the structural analysis of the binding of fragments of potential drug molecules to target proteins (Murray & Blundell, 2010 ▶). Although X-ray-based screening is slower than other fragment-screening approaches, it has the advantage, once a robust crystallization system has been established, of providing relatively high-resolution structural information on protein–ligand complexes. This information can then be used in a skilful procedure to design potential lead compounds which can help to drive novel drugs to clinical trials with reduced rates of attrition. Fragment-based drug design thus has long-term potential returns in terms of drug optimization and, apart from the emergence of companies which use this technique as a core method in drug discovery (*e.g.* Astex Pharmaceuticals Inc.; http://www.astx.com/technology), most pharmaceutical companies now have some fragment-based projects which require X-ray diffraction data from very large numbers of samples. Only synchrotron-based MX beamlines combine the X-ray beam characteristics (*i.e.* high intensity, micro-beam, microfocus) necessary in many cases to obtain diffraction data to a resolution at which fragment-binding modes are interpretable with the HTX environment (short exposure times, fast-readout detectors, rapid sample changers) required for an effective fragment-based drug-design process.

## 3. Standardization and automation

Industrial access to the ESRF’s MX beamlines takes two main forms: experimenters carry out their own synchrotron data-collection experiments, either at the synchrotron facility itself or from their home laboratory *via* remote control; synchrotron data-collection experiments are outsourced to the ESRF *via* the MXpress data-collection service. Both forms of access require robust and reliable technology in order to ensure that beam time is used efficiently and, particularly when data collections are subcontracted out to ESRF staff, to provide value for money.

To facilitate robustness and reliability, the experimental environments of all of the ESRF MX beamlines, regardless of their X-ray beam characteristics, have been standardized (see Leonard *et al.*, 2007 ▶; de Sanctis *et al.*, 2012 ▶). Standardization has imposed common features on all of the beamlines, with the same or similar hardware and software present everywhere; this makes maintenance and software support easier. Moreover, the fact that all the beamlines have the same general components means that it is easy for the user to adapt to the experimental environment, be it on a high-throughput tuneable beamline, a fixed-wavelength beamline or a microfocus beamline: the key elements of the beamline stay the same and only the type of experiment being attempted changes. Thus, as users become familiar with the ESRF hardware and software environment they are left to focus only on the experiment in hand rather than on differences in hardware or software.

Although the development of standardized and automated processes over a number of varied beamlines can initially be slower than developing a specific feature for a single beamline, the final system is easier to maintain and gives an enhanced, more user-friendly environment. In addition, once the automation of processes has been established and shown to be reliable, they are easy to control remotely. In this way, remote control of the ESRF MX beamlines was relatively easy to implement once automatic control of the beamlines themselves had been put into place (Gabadinho *et al.*, 2008 ▶, 2010 ▶).

## 4. The requirements of a data-collection service

**Figure 1:**
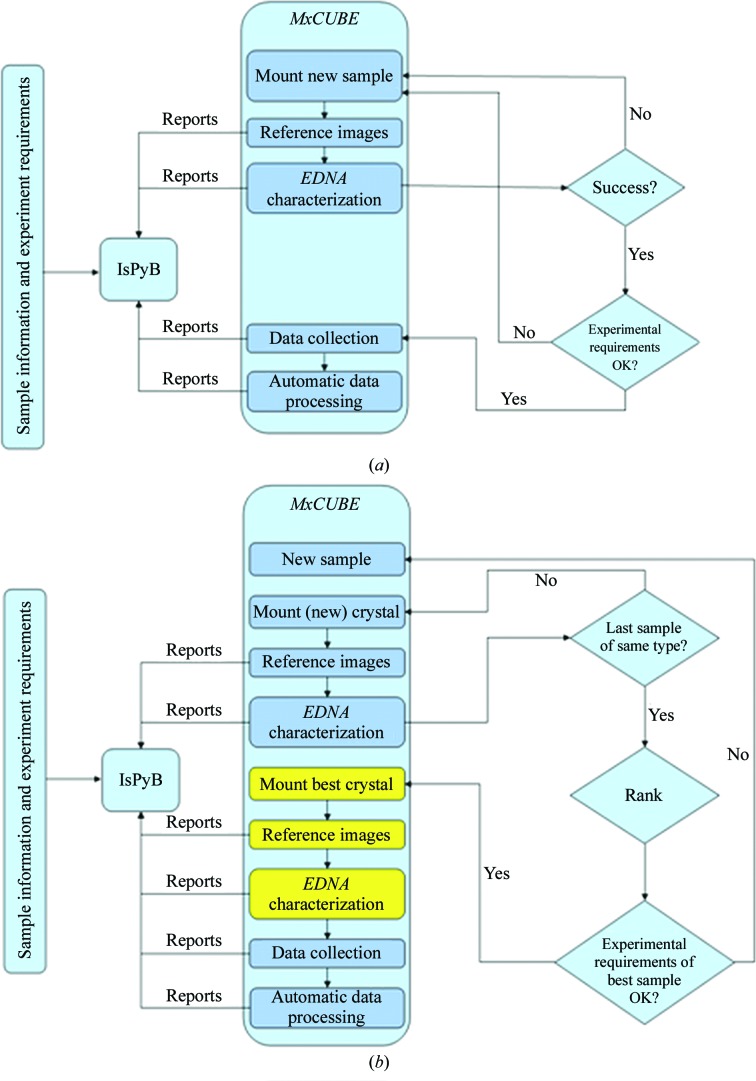
Workflows used by the MXpress data-collection service. (*a*) The workflow used when only a single crystal of each sample is provided. (*b*) The workflow used when more than one crystal of each sample is provided and ranking of diffraction characteristics is used to choose the best sample for full data collection. In both cases *EDNA*/*BEST* (Incardona *et al.*, 2009 ▶; Bourenkov & Popov, 2010 ▶) is used for the characterization of diffraction properties and the calculation of data-collection strategies.

**Figure 2:**
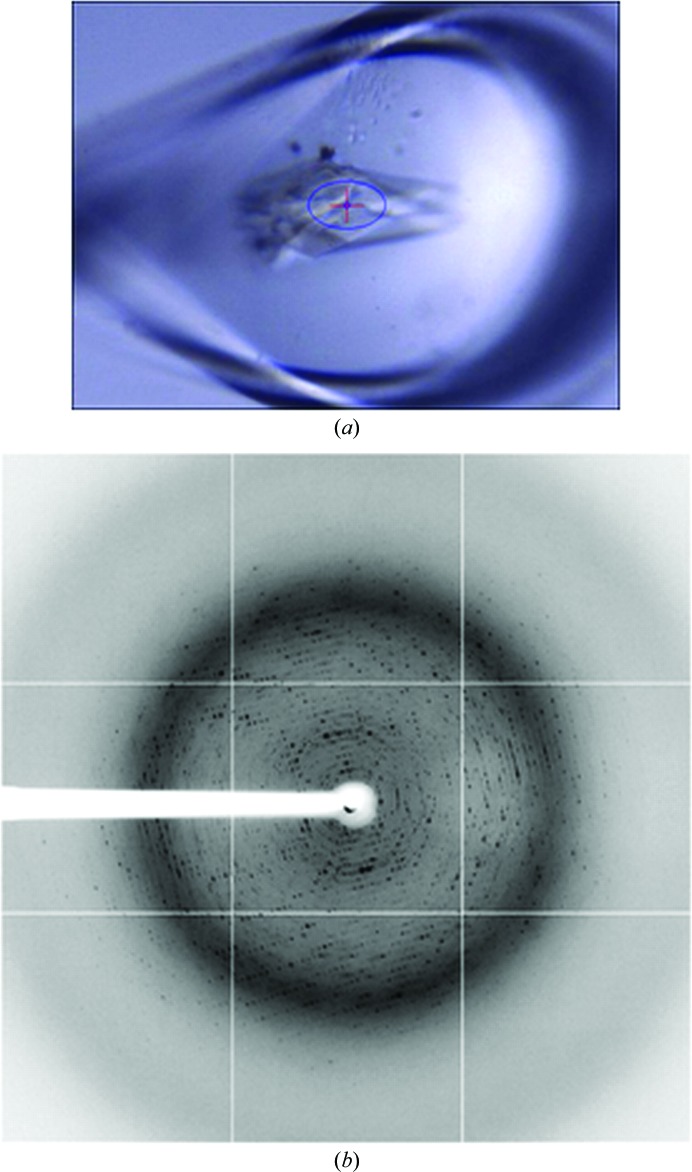
Good-quality diffraction data sets from crystals exhibiting poor-quality diffraction. (*b*) shows a test diffraction image collected from the crystal shown in (*a*).

The MXpress data-collection service at the ESRF was established in 2002, initiated by a few isolated requests for data collections for a small number of samples. However, it soon became clear that several other groups wished to outsource a large proportion of their data-collection needs to the ESRF and that if the ESRF was to be able to cater for such a demand then a dedicated infrastructure was required. Particular concerns of both clients and of the ESRF staff were to ensure that, for any given sample, the best data-collection strategy was employed and that provision was made for the tracking of samples to, from and during their time at the ESRF. To address the former concern, the MXpress service drove and now makes extensive use of sample-characterization and data-collection strategy software (Fig. 1 ▶). To address the latter concern, the ESRF was at the forefront of the development of a LIMS combining sample tracking and experiment reporting during synchrotron-based MX experiments. The result, ISPyB, is streamlined and robust, includes the possibility of communicating experimental requirements (for each individual sample), facilitates the following of the progress of data-collection sessions remotely and allows full and fast interaction between clients and the ESRF staff carrying out the data collection. The latter point was particularly important in the early days of the data-collection service. While the ESRF has been at the forefront of collaborations such as *DNA* (Leslie *et al.*, 2002 ▶) and *EDNA* (Incardona *et al.*, 2009 ▶) aimed at developing software which matches the best data-collection strategy to the sample and the beamline on which an experiment is being performed, feedback from industrial clients highlighted the fact that different projects have different data-quality requirements. For example, some ligand-soaked crystals can produce diffraction patterns of very poor quality, but full data diffraction data sets collected from these crystals can provide perfectly acceptable electron-density maps with interpretable ligand density for the intended application (Fig. 2 ▶; Table 1 ▶). Initial implementations of *DNA* and *EDNA* tended to categorize samples with poor diffraction patterns as being unsuitable for data collection, usually because of the failure of automatic autoindexing procedures. Modifications made to the pipelines involved mean that this is no longer the case, a development that has benefited both academic and industrial users of the ESRF’s MX beamlines.

## 5. The beamline database ISPyB

**Figure 3:**
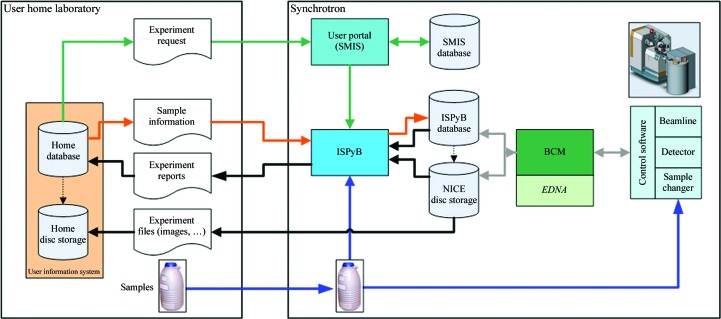
The application environment and data flow for the ISPyB database. The user’s home laboratory is on the left of the figure and the synchrotron facility is on the right. Details of experimental session scheduling are retrieved by ISPyB from the ESRF user portal database (Scientific Management Information System; SMIS). Prior to each experimental session, information (protein name, organism, safety class, diffraction plan *etc.*) concerning the samples to be studied can be provided to the ISPyB database and can be downloaded to the beamline-control module (BCM) on the day of the experiment in order to guide data collection. During an experiment ISPyB records, stores and makes available to users in real time all relevant experimental parameters for each data collection as well as the results of automatic sample evaluation using *EDNA* and automatic data processing.

The need, alluded to above, of industrial clients of the ESRF’s MX beamlines for robust sample tracking and experiment reporting was one of the driving forces which led to the development and deployment at the ESRF in 2001 of PXWeb, a prototype LIMS combining sample tracking and experiment reporting during synchrotron-based MX experiments (Arzt *et al.*, 2005 ▶). As both academic and industrial use of the ESRF’s MX beamlines has expanded, information exchange between the home laboratory and the synchrotron became critical and an upgraded LIMS, ISPyB, was produced (Fig. 3 ▶). A recent publication describes both the current status of ISPYB and its functionality (Delagenière *et al.*, 2011 ▶).

Perhaps the most basic function of ISPyB is the production of bar-coded dewar labels for the shipment of transport dewars to the ESRF. The labels include the names of the laboratory contact and ESRF local contact as well as the date of the experiment and the beamline. All of this information is required in order to ensure that the dewar arrives at the right beamline on the right day. Labels for the return shipment, including courier return details, can also be produced for use by ESRF staff.

Prior to an experimental session ISPyB allows all pertinent information to be provided for each sample to be studied to the experimenters, whether they be colleagues or ESRF staff. A minimal set of information includes acronym, sample name, puck identity, puck position and comments concerning data-collection priority. A fuller set of information includes a so-­called ‘diffraction plan’ and includes the space group (if known), unit-cell dimensions, required resolution, acceptable resolution and required multiplicity. Further comments may also be uploaded, for example if the crystal is known to diffract anisotropically. On the day of the experiment, logging into the beamline-control module *MxCuBE* with the relevant experiment number causes all information to be displayed in the *MxCuBE* GUI, where it can be used to drive sample loading/unloading, the creation of the directory path and image nomenclature or the execution of a data-collection pipeline (Beteva *et al.*, 2006 ▶).

During data collections ISPyB provides an easily accessible tool which clients at home in their laboratory can use to monitor progress in real time. Once connected to the relevant data-collection session in ISPyB *via* a web browser, a top-level chronological summary provides basic information concerning the individual sample data collections that have already been carried out or that are in progress. This basic information is updated to include a summary of data-processing statistics from the ESRF’s autoprocessing system (Monaco *et al.*, 2012 ▶) as and when this becomes available. More detailed information regarding experimental and beamline parameters, sample characterization and data-collection strategies calculated using the program *BEST* (Bourenkov & Popov, 2010 ▶) as implemented in *EDNA* (Incardona *et al.*, 2009 ▶; see below) and the full results of automatic data processing can be accessed by selecting a particular data collection and clicking to a lower level. Here, snapshots of the sample and diffraction patterns are displayed. It is also possible to directly download files containing the processed diffraction data. The top-level data-collection summary is available in various formats, including a Portable Document Format (PDF) report. This is particularly important for the MXpress data-collection service, as this report is used to confirm to clients the number of samples processed and hence the amount to be invoiced.

## 6. The power of *EDNA*

Collecting data from crystals of biological macromolecules at synchrotron beamlines involves the optimization of a number of parameters that are required for the collection of a data set that is fit for purpose (for example, to a specified completeness, accuracy and maximum resolution) while at the same time avoiding the pitfalls of radiation damage, overloaded reflections, poorly measured reflections and overlaps (Dauter, 2010 ▶). The decision making undertaken to produce an optimized strategy may be influenced by factors including the time available, the sample performance, the beamline setup *etc.* Data are collected to answer a specific biological question and different applications require that certain characteristics of the data are optimized. Searching for protein-bound ligands in electron-density maps does not in principle require high levels of data completeness or accuracy (Kleywegt, 2007 ▶; Dauter, 2010 ▶). However, the chances of identifying weakly bound ligands or fragments are only improved if crystallographic data are complete, accurate and to reasonably high resolution.

When establishing the MXpress data-collection service a policy decision was made that in the absence of instructions to the contrary, if a sample/diffraction pattern could be indexed and the diffraction was the same or better than the required resolution communicated in the ‘diffraction plan’ (see above) then the best possible resolution data set with a multiplicity of ∼4.0 would be collected using a data-collection strategy calculated by *DNA* (Leslie *et al.*, 2002 ▶). As the radiation damage of cryocooled crystals at third-generation synchrotron sources has been well documented (see, for example, Garman & McSweeney, 2007 ▶), the total exposure time allowed for a data collection was hardwired into the system and based on empirical observation. Nowadays, this procedure is somewhat more sophisticated. *DNA* has been replaced by a more modular workflow tool, *EDNA* (Incardona *et al.*, 2009 ▶), which, like *DNA* before it, sequences the running of all programs required for data-collection strategy calculations: *MOSFLM* (Leslie, 2006 ▶) for autoindexing and integration of test images, *RADDOSE* (Paithankar & Garman, 2010 ▶) for calculation of allowed total exposure times for data collections and *BEST* (Bourenkov & Popov, 2010 ▶) for the calculation of the data-collection strategy itself. Additionally, user estimates of the crystal size and chemical composition, together with accurate measurements of the photon flux at the time that the test images were collected, are passed to *EDNA* by the *MxCuBE* GUI for use in *RADDOSE*. The results of all strategy calculations are subsequently passed to ISPyB for storage and display. This allows the ranking of diffraction characteristics, notably crystal mosaicity and diffraction limit, and thus the choice of the best crystal for subsequent data collection.

## 7. Fast-readout pixel detectors and screening by data collection

The use of *EDNA* is particularly helpful when collecting diffraction data using the PILATUS 6M pixel detector, as limitations of current image-visualization software means that it is difficult to manually determine the diffraction limits.

**Figure 4:**
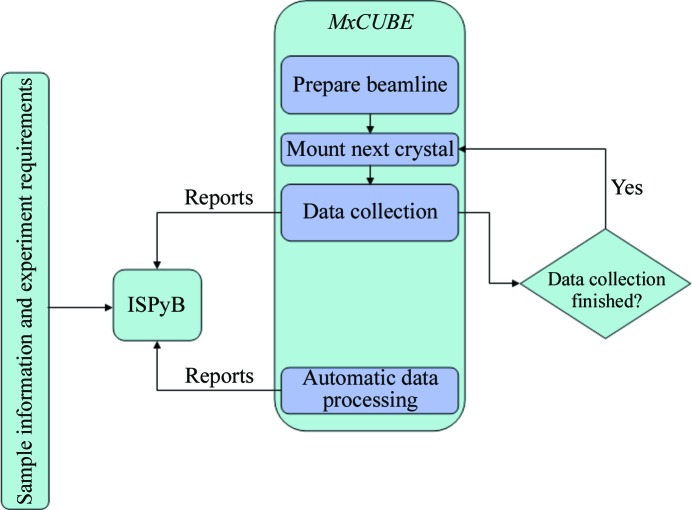
The workflow used in ‘screening by data collection’. Here, the same data-collection strategy is used for all samples and the results of automatic data processing are used to choose the best data set(s) for use in subsequent downstream analyses.

The fast readout (∼2 ms) times and high framing rates of the current generation of pixel detectors, coupled with shutterless data-collection protocols (Hülsen *et al.*, 2006 ▶), means that the collection of complete X-ray diffraction data sets may be achieved very quickly even when using the fine-ϕ-sliced ‘oscillations’ recommended for such detectors (Mueller *et al.*, 2012 ▶). On ESRF beamline ID29, 1800 PILATUS 6M 0.1° ‘oscillation’ images are routinely collected in 1.25 min. Potential data-set throughput is therefore very high on beamlines equipped with such detectors. However, potential throughput is reduced dramatically by calculating data-collection strategies before data collection. Perhaps a more efficient use of beamlines that are equipped with such detectors would be a return to the so-called ‘American method’, suggested as long ago as 1983 (Rossmann & Erickson, 1983 ▶), of ‘shoot first and ask questions later’ and ‘standard experiments’ to collect data sets using a standard strategy that takes into account crystal lifetime in the X-ray beam, using the results of automatic data processing to choose the best data set for use in subsequent analyses. At the suggestion of some of our industrial clients, such a ‘screening by data collection’ has been implemented on ID29 at the ESRF and operates using the PILATUS 6M at 25 Hz framing rates in a semi-automatic pipeline mode (Fig. 4 ▶).

One of the disadvantages of working in such a ‘screening by data collection’ fashion is that a significant fraction of the data which are collected are ultimately not used: time and disk space may be wasted backing up and saving many gigabytes of undesirable data. The solution here is to rapidly assess whether data are useful before transferring them to the home laboratory or spending time making backups. Perhaps the easiest way of doing this is to see whether the data are fit for purpose, which, given today’s high-quality data-analysis and structure-solution software, is an entirely plausible notion. It is thus a priority for the ESRF to provide access *via* ISPyB in the near future to rapid, robust and reliable structure-solution pipelines that can be used to validate the usefulness of the collected data. As a first step towards this, we have implemented a rapid automatic data-system package for the integration and analysis of diffraction data sets. Further details of this will be published elsewhere (Monaco *et al.*, 2013 ▶).

## 8. The advantages of working with industrial clients

The interaction between the ESRF and its industrial clients has advantages for both parties: improved (relative to in-house sources) X-ray diffraction data provide an impetus for many projects, whether collected by the clients themselves (see, for example, Bax *et al.*, 2010 ▶; Ward *et al.*, 2011 ▶) or using the MXpress data-collection service (see, for example, Keil *et al.*, 2011 ▶; Liu, Chen *et al.*, 2011 ▶); the income generated is reinvested into improving the existing facilities and providing extra staff. However, perhaps the major advantage for the ESRF of working with industrial clients is that they are very quick to embrace new technology and automation if they are convinced that developments in these areas can help increase both throughput and value for money. Once a particular technology has been embraced, industrial clients are ready to invest in the appropriate equipment and their high-throughput demands then provide the best sort of stress test for the technology deployed. Industrial clients are also not afraid to provide critical feedback when technology does not perform as advertised, to suggest how prototype technology might be improved or how currently available technology can be best deployed to their, and by implication academic users’, best advantage.

**Figure 5:**
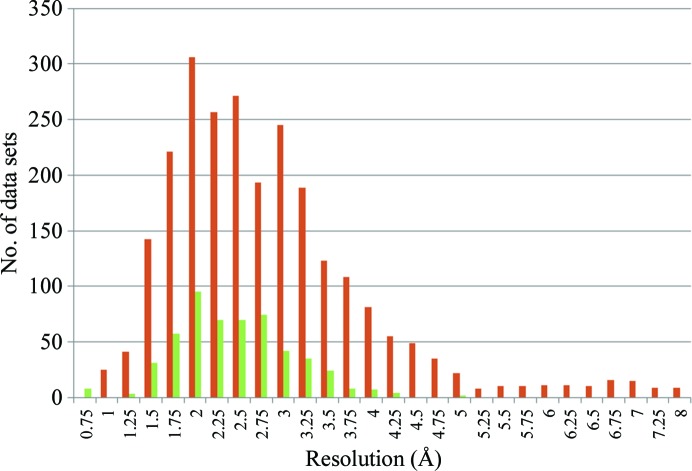
A comparison, based on the results of automatic data processing covering a period of around 12 months on all of the ESRF MX beamlines, of the resolution of complete diffraction data sets collected on the ESRF MX beamlines by academic (orange bars) and industrial (green bars) users. While the maxima of both distributions is at a *d*_min_ of ∼2 Å, that for academic users shows a much longer tail extending to a *d*_min_ of ∼8 Å. The few very high resolution (*d*_min_ of ∼0.75 Å) data sets collected by/for industrial users result from data-collection experiments on crystals of small molecules.

Industry has a very well structured project organization, exploiting robust crystallization systems which can withstand tough soaking conditions and the need for medium- to high-resolution diffraction data (Fig. 5 ▶) with a multiplicity of 3–4. This framework has helped to drive the development of hardware and software for the ESRF beamlines which ensures reliable, routine and rapid MX data collection that can be used with equal facility by experimenters themselves or by ESRF staff carrying out service data collections. Nevertheless, it is clear that the automation of more complex experiments is also a priority at the ESRF. The future of ESRF MX beamlines thus lies in developing protocols for more complex experiments, including multi-positional data collections from areas of crystals identified using diffraction cartography experiments (Bowler *et al.*, 2010 ▶) and the merging of several isomorphous complete or partial data sets to provide diffraction data that are fit for purpose (Ji *et al.*, 2010 ▶; Liu, Zhang *et al.*, 2011 ▶; Giordano *et al.*, 2012 ▶).

Industrial use of the resources of the ESRF’s Structural Biology Group is not confined to its MX beamlines. Pharmaceutical companies are increasingly using the small-angle X-ray scattering study of biological molecules in solution (BioSAXS) as a predictive tool for crystallization (see, for example, Vivarès & Bonneté, 2004 ▶; Geerlof *et al.*, 2006 ▶), in drug formulation (see, for example, Mackeben & Müller-Goymann, 2000 ▶), as a complementary method for the structural characterization of large macromolecular complexes (see, for example, Putnam *et al.*, 2007 ▶; Mertens & Svergun, 2010 ▶) and in the monitoring of ligand binding in solution (see, for example, Riek *et al.*, 2008 ▶; Forstner *et al.*, 1998 ▶). Based on the experience gained on its MX beamlines, the ESRF has constructed a highly automated facility for such studies and, in collaboration with EMBL Hamburg and Diamond Light Source, is constructing BioSAXS data-collection and processing pipelines. Included in these developments is a modified version of the ISPyB database for sample tracking, experiment logging and the automatic collection and treatment of BioSAXS scattering curves. As for the developments deployed on the ESRF MX beamlines, such pipelines will be of benefit to academic and industrial experimenters alike.

## 9. Conclusions

For more than fifteen years, the ESRF has provided data-collection facilities and services for the pharmaceutical industry that are based on a portfolio of beamlines with a wide range of functionality and beam characteristics. The inter­action between the ESRF and its MX industrial clients has been, and continues to be, mutually beneficial. The willingness of industrial clients to embrace automation and their requirements for reliable robust high-throughput experimental environments have been significant driving forces in the development and refinement of much of the functionality of the ESRF’s macromolecular crystallography resources. This enhanced framework is now taken for granted by both industrial and academic users alike and is now providing the basis for the ‘second-generation automated beamlines’ for macromolecular crystallography being constructed as part of the ESRF’s Upgrade Programme.

## Figures and Tables

**Table 1:** The results of automatic data processing (Monaco *et al.*, 2012 ▶) for a data set collected using a data-collection strategy calculated by *EDNA*/*BEST* and based on the test image shown in Fig. 2 ▶

Space group	*C*222
Unit-cell parameters (Å)	*a* = 145.7, *b* = 18.21, *c* = 89.7
Resolution range (Å)	100–2.71 (2.90–2.71)
Observed reflections	144887 (26978)
Completeness (%)	99.6 (99.6)
Multiplicity	4.41 (4.53)
〈*I*/σ(*I*)〉	12.3 (3.12)
*R*_merge_ † (%)	10.4 (62.4)

† *R*_merge_ = 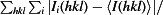 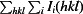, where *I_i_*(*hkl*) is the *i*th observation of reflection *hkl* and 〈*I*(*hkl*)〉 is the weighted average intensity for all observations of reflection *hkl*.
